# Case report: Mild phenotype of a patient with vascular Ehlers–Danlos syndrome and *COL3A1* duplication mutation without alteration in the [Gly-X-Y] repeat sequence

**DOI:** 10.3389/fgene.2022.1017446

**Published:** 2022-11-18

**Authors:** Shujiro Hayashi, Tomomi Yamaguchi, Tomoki Kosho, Ken Igawa

**Affiliations:** ^1^ Department of Dermatology, Dokkyo Medical University School of Medicine, Mibu, Japan; ^2^ Department of Medical Genetics, Shinshu University School of Medicine, Matsumoto, Japan; ^3^ Center for Medical Genetics, Shinshu University Hospital, Matsumoto, Japan; ^4^ Division of Clinical Sequencing, Shinshu University School of Medicine, Matsumoto, Japan; ^5^ Research Center for Supports to Advanced Science, Shinshu University, Matsumoto, Japan

**Keywords:** Vascular Ehlers–Danlos syndrome, COL3A1, triplet repeat sequence, endoplasmic reticulum stress, unfolded protein response, in-frame mutation

## Abstract

**Background:** Vascular-type Ehlers–Danlos syndrome (vEDS) is an autosomal dominant inherited disorder caused by a deficit in collagen III as a result of heterogeneous mutations in the α1 type III collagen gene (*COL3A1*). Patients with vEDS often experience the first major complications in their early 20s and >80% have at least one complication by their 40s, reducing their average life expectancy to 48 years. Most commonly, vEDS variants are heterozygous missense substitutions of a base-pair encoding a glycine (Gly) residue of the [Gly-X-Y] repeat of the *COL3A1* protein. When a peptide chain derived from a mutant allele is present in the procollagen triple helical structure, the helical structure cannot be maintained. Therefore, typically, the mutated collagen peptide induces a dominant negative effect on procollagen production. We reported the case of a patient with vEDS and a unique novel duplication mutation without alteration in the [Gly-X-Y] triplet repeat sequence.

**Case presentation:** A 58-year-old man developed a sudden disorder of consciousness and abdominal pain and was consequently taken to a nearby hospital, where an intra-abdominal aneurysm was found, in addition to mild small joint hypermobility and acrogeria. There has been no history of spontaneous pneumothorax, dislocation, or subcutaneous hematoma. The analysis of genomic DNA from a blood sample identified a likely pathogenic in-frame duplication mutation in the *COL3A1* gene coding region. Interestingly, this mutation is not expected to alter the [Gly-X-Y] triplet repeat sequence. We verified the mutation’s pathogenicity by performing an analysis of synthetic procollagen from cultured skin fibroblasts, electron microscopy, and mRNA expression analysis of unfolded protein response sensors for endoplasmic reticulum (ER) stress.

**Conclusion:** Although the clinical findings of the case were mild, when compared to typical vEDS, decreased α1 collagen III levels and morphological abnormalities of the collagenous bundles were observed in the patient samples when compared with the normal control samples. Our evidence supports the conclusion that this variant is pathogenic. However, unlike the common vEDS, ER stress was not observed, and the mild phenotype presentation was suggested to be due to the unique mutation, allowing the triple helical structure to be maintained to a certain extent.

## Introduction

Vascular-type Ehlers–Danlos syndrome (vEDS) is an autosomal dominant inherited disorder with a frequency of 1:100,000–250,000 (Byers et al., 2017). vEDS is caused by a deficit in collagen III that results from heterogeneous mutations in the α1 type III collagen gene (*COL3A1*). The reduction in collagen III can affect the hollow organ walls, such as those of the uterus, intestines, and medium- and large-sized arteries, and the fragility of the connective tissues. In addition to various characteristic manifestations, such as translucent skin, easy bruising, characteristic facial appearance, small joint hypermobility, acrogeria, and others, vEDS patients occasionally also experience fatal complications, such as macrovascular rupture, intestinal perforation, and uterine rupture during pregnancy (Shimaoka et al., 2010; Malfait et al., 2017). Patients with vEDS often experience their first major complication in their early 20s and >80% of them have at least one complication by their 40s, reducing their average life expectancy to 48 years (Pepin et al., 2000).

Collagen proteins comprise a triple helical structure from three peptide chains, and this distinctive structure provides strong stability to the protein. To achieve a triple helical structure, three rich amino acids, namely, glycine (Gly), proline, and modified proline called hydroxyproline, are required. Gly should be one of the three consecutive amino acids to maintain the triple helical structure (Kramer et al., 1999). Most commonly, vEDS variants are heterozygous missense substitutions at a Gly-coding residue in the context of [Gly-X-Y] repeats, and secondly, vEDS are splice-site mutations in *COL3A1* (Frank et al., 2015). The mutant collagen peptide induces a dominant negative effect on procollagen production. Procollagen is organized as a triple helical structure formed by three peptide chains (Frank et al., 2015). When a peptide chain derived from the mutant allele is present in the procollagen triple helical structure, the helical structure cannot be maintained (Malfait et al., 2017). The expression level of collagen III produced by fibroblasts of the patients is extremely low, approximately 10%–20% when compared to normal healthy individuals (Shimaoka et al., 2010).

Here, we report the case of a patient with vEDS and a unique novel duplication mutation without alteration in the [Gly-X-Y] triplet repeat sequence. This case shows a mild phenotype, suggesting that the triple helical structure of collagen III chains may have been maintained with this particular mutation to some extent.

## Case presentation

**FIGURE 1:**
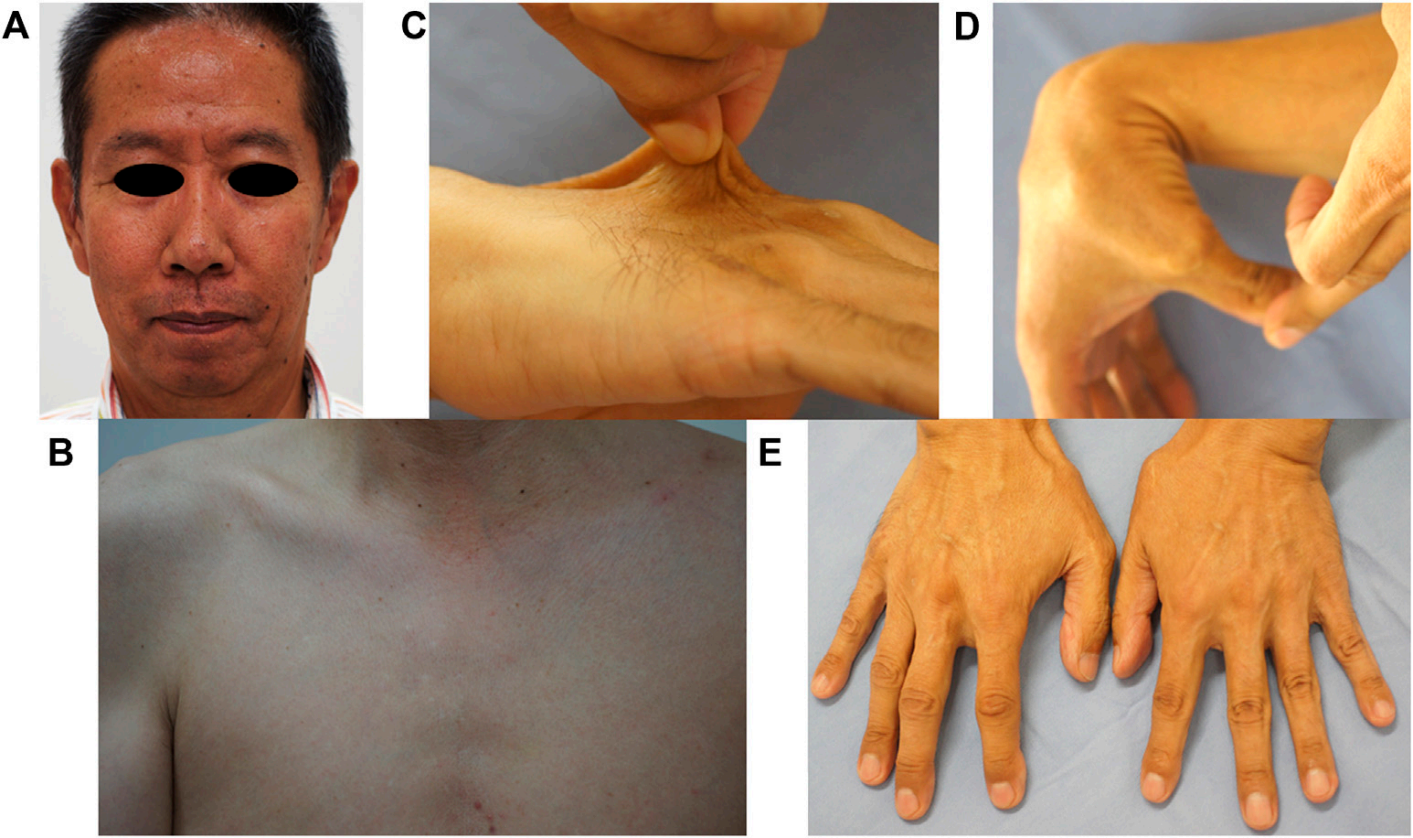
Clinical findings. No remarkable findings in terms of vEDS facial features **(A)**, no permeability of subcutaneous blood vessels **(B)**, and no skin hyperextension **(C)** were observed. Mild small joint hypermobility **(D)** and acrogeria were observed **(E)**.

At presentation, a 58-year-old man developed a sudden disorder of consciousness and abdominal pain and was taken to a nearby hospital, where multiple intra-abdominal aneurysms were found. The aneurysm was under control owing to the administration of antihypertensive drugs, but the patient was referred to our facility because of suspected hereditary connective tissue disease due to the presence of joint hypermobility. The patient’s parents are deceased and had not undergone genetic testing prior to death. Upon patient interview, it was revealed that neither parent had any symptoms attributed to vEDS. Furthermore, informed consent was obtained from the patient for the publication of clinical photographs. No remarkable vEDS facial feature, subcutaneous blood vessel permeability, or skin hyperextension was observed (Figures 1A–C). Contrarily, mild small joint hypermobility and acrogeria were observed (Figures 1D,E). The patient had no history of spontaneous pneumothorax, dislocation, or subcutaneous hematoma. Additionally, no known relatives, including his two daughters, had symptoms of suspected vEDS. The in-frame duplication mutation in the *COL3A1* gene was identified through the sequencing analysis of genomic DNA from the blood sample (see below).

The patient was suspected of having vEDS; however, the onset at old age and good clinical course presentation are atypical for vEDS. Thus, we proceeded with the verification of the pathogenicity of the gene mutation identified in this case after obtaining an informed consent from the patient.

After the analysis, the patient understood that the symptoms were probably due to vEDS. The patient had two asymptomatic daughters in their 20s. It is a given that the identified mutation could lead to a mild phenotype if inherited and symptoms could develop in his daughters in the future even if they have been currently asymptomatic. The patient was informed regarding the importance of genetic testing for his daughters, but they declined to undergo the recommended evaluation. During the clinical course, the prescribed antihypertensive drug was switched to celiprolol, and no serious complications occurred during the 8 years after the diagnosis.

## Methods

Determination of the base sequence on the genomic DNA extracted from the blood samples was performed using the Sanger sequencing method as previously reported (Shimaoka et al., 2010). Observation of the tissues obtained by skin biopsy with transmission electron microscopy (TEM) and real-time reverse-transcription polymerase chain reaction (RT-PCR) was performed according to a previously reported protocol (Ishikawa et al., 2021). The analysis of synthetic procollagen from cultured skin fibroblasts was conducted according to a protocol described elsewhere (Shimaoka et al., 2010). Briefly, dermal fibroblasts were cultured to confluence in 100 × 20-mm dishes in DMEM containing 10% FBS. Furthermore, the fibroblasts were incubated with DMEM containing 1% FBS and 5 μCi·ml^−1^ of 2,3-[3 H] proline in the presence of 50 μg·ml^−1^ of L-ascorbic acid 2-phosphate for 24 h. The labeled proteins secreted into the culture medium were precipitated by the addition of 5% trichloroacetic acid, and the precipitate was dissolved in 0.05 mol·L^−1^ acetic acid and digested with pepsin. Moreover, the labeled proteins were separated using sodium dodecyl sulfate–polyacrylamide gel electrophoresis in the presence or absence of 2-mercaptoethanol. The radioactive bands were detected by fluorography. We compared these samples with the samples of a single normal control (a 65-year-old man) and a vEDS disease control (a 53-year-old man having c.3365 + 1G>A in *COL3A1* in genome DNA), who were matched by age and sex. The following sequences reported in the past were used as the primers used in real-time RT-PCR (Li et al., 2016). *ATF6B*: forward 5′-GAG​TCA​TCG​CGT​CTC​TCC​AC, reverse 5′-GGC​CTC​AGA​GTT​GAC​GGA​AG, *CHOP*: forward 5′-AAG​GCA​CTG​AGC​GTA​TCA​TGT, reverse 5′-TGA​AGA​TAC​ACT​TCC​TTC​TTG​AAC​A, and *GAPDH*: forward 5′-GGC​CTC​CAA​GGA​GTA​AGA​CC-3′, reverse 5′-CTG​TGA​GGA​GGG​GAG​ATT​CA-3′.

Tukey’s test was implemented for the statistical analysis of the real-time RT-PCR data. *p*-values of ≤0.05 were considered significant. The data are presented as the mean ± standard error of the mean. Statistical comparisons were performed using the Statistical Package for the Social Sciences version 18 (SPSS, Inc., Chicago).

## Results

### Results of the genetic analysis point to an in-frame duplication in the α1 type III collagen gene as the source of vascular-type Ehlers–Danlos syndrome pathology

**FIGURE 2:**
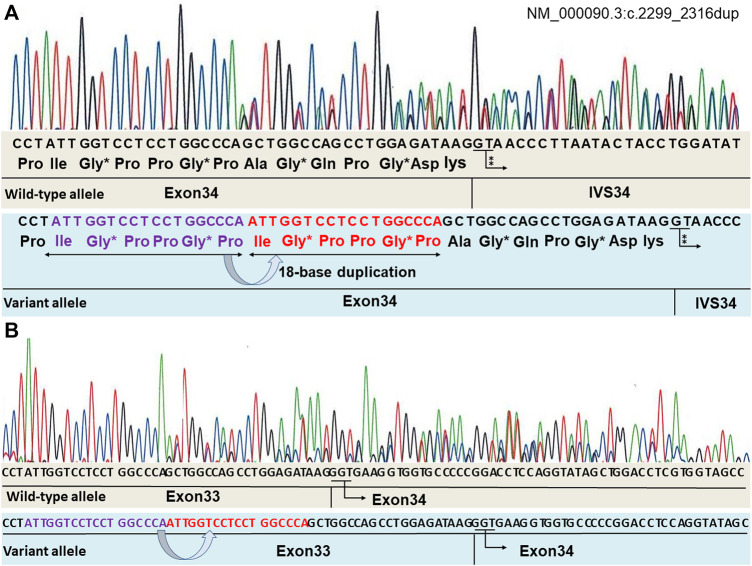
Mutation analysis of the *COL3A1* genomic DNA identified a variant (NM_000090.3:c.2299_2316dup). The repeating sequence of [Gly-X-Y] is retained in the variant allele. “*” indicates Gly positioned in a triplet repeat sequence. “⁑” indicates GT, indicating the beginning of the intron **(A)**. RNA sequencing is shown in **(B)**. mRNA obtained from the fibroblasts of the patient was converted to cDNA using reverse transcriptase, and Sanger sequencing analysis of the cDNA was performed. There are no abnormal nucleotides in the border of exons 33–34. Both alleles have the normal subsequently transcribed exon 34 without an exon skip.

By performing Sanger sequencing on the patient’s genome DNA, the [heterozygous] mutation (NM_000090.3:c.2299_2316dup (p.Ile767_Pro772dup) was detected in the *COL3A1* gene, encoding α1 type III collagen. This 18-bp in-frame variant maintained the common collagen motif of the [Gly-X-Y] triplet repeat sequence (Figure 2A). This mutation was not registered in the dbSNP of the National Center for Biotechnology Information and the gnomAD. The mutation was predicted as “deleterious” on the basis of the *in silico* pathogenic prediction using MutationTaster. However, the pathogenicity of this in-frame variant could not be assessed by annotation with the others in multiple *in silico* programs. Therefore, it was difficult to determine the pathogenicity based only on the genetic analysis.

To address the possibility of this duplication mutation leading to a splicing anomaly, the mRNA obtained from the fibroblasts of the patient was converted to cDNA using reverse transcriptase, and Sanger sequencing analysis of the cDNA was performed. Evidence for the presence of a splicing variant was not found (Figure 2B).

### Results of the collagen analysis suggest that the mutation affects α1 type III collagen gene protein expression

**FIGURE 3:**
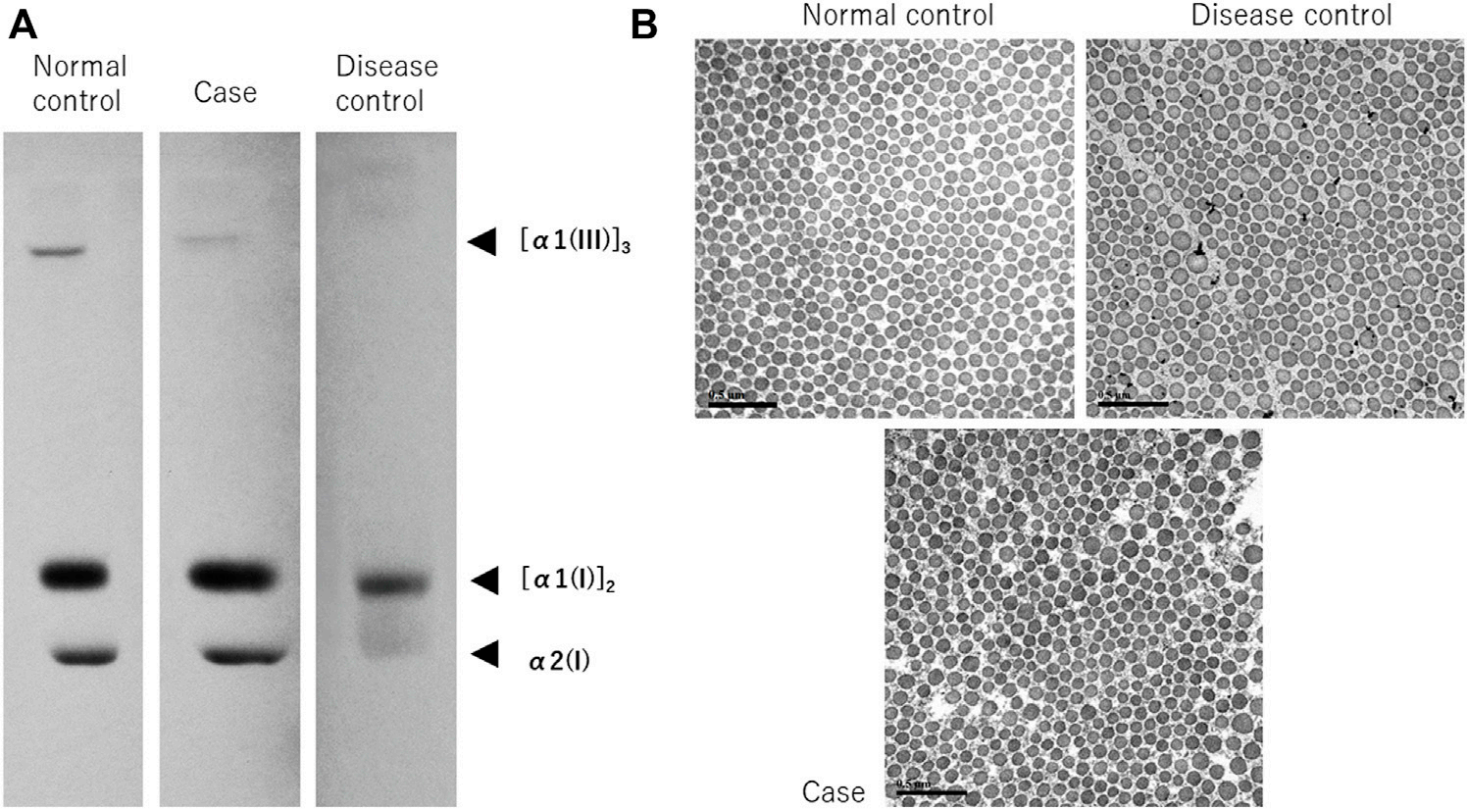
Synthetic analysis of procollagen and electron microscopy of collagenous bundles. Procollagen from cultured skin fibroblasts was electrophoresed and analyzed **(A)**. In the disease control, α1 collagen III is barely visible, but in the normal control, it is apparent. Contrarily, the expression in this case’s sample is at an intermediate level between the normal and disease control samples, suggesting a deficiency in expression. Observation of collagenous bundles under TEM **(B)**, bar; 0.5 µm). Numerous small collagenous bundles were found in the disease control, and as a result, the size difference was conspicuous. However, the size was similar to that of the normal control. In the present case, the size difference was milder than it was in the disease control, owing to the structural abnormalities observed when compared with the normal control.

Procollagens from the cultured skin fibroblasts were electrophoresed and analyzed using our previously reported methods (Shimaoka et al., 2010). vEDS is caused by the inhibition of the synthesis of functional proteins due to a dominant negative effect. The synthesis of collagen III is predicted to be extremely low in patients with vEDS. As expected, in the disease control, α1 collagen III was barely visible, although it was apparent in the normal control. Contrarily, the expression of α1 collagen III in the patient’s sample was at an intermediate level between those of the normal and disease control samples, demonstrating a deficiency in expression (Figure 3A).

### Collagenous bundles show intermediate morphology between typical vascular-type Ehlers–Danlos syndrome and healthy samples

TEM showed numerous small collagenous bundles in the disease control, and as a result, the size difference was conspicuous. However, the size was similar to that of the normal control. This finding is in line with the characteristic findings in vEDS (Smith et al., 1997; Ishikawa et al., 2021). Contrarily, in the present case, the degree of size difference in the collagenous bundles was lesser than it was in the disease control, but the collagenous bundles were observed to exhibit structural abnormalities when compared with those of the normal control (Figure 3B).

### Endoplasmic reticulum stress is not apparent in the case

**FIGURE 4:**
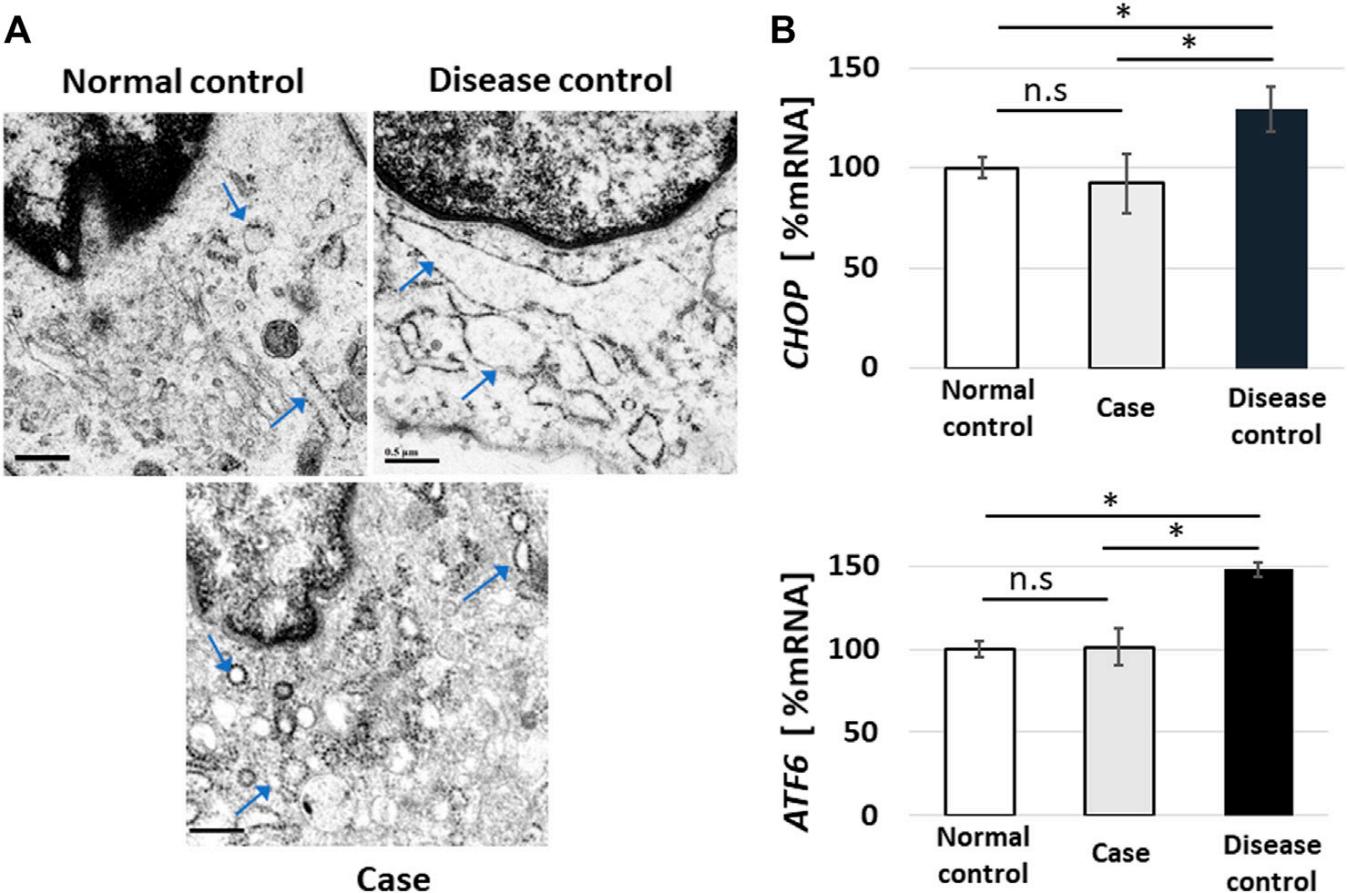
Endoplasmic reticulum (ER) stress and real-time reverse-transcription polymerase chain reaction (RT-PCR) for unfolded protein response sensors. Fibroblasts showed no dilation of the ER in the normal control, but images showing dilated fibroblasts of the ER were obtained in the disease control. In the present case, the expansion of the ER was not noticeable (arrowheads indicate ER **(A)**; bar, 0.5 µm). In real-time RT-PCR using mRNA extracted from cultured fibroblasts, PCR was performed on four technical replicates of each mRNA sample, and the results were averaged. *ATF6* and *CHOP* were highly expressed in the disease control when compared to the normal control; however, their expression levels in the present case were almost the same as those of the normal control **(B)**, error bar: standard error, ns: not significant, *p < 0.05).

Endoplasmic reticulum (ER) stress is a state in which abnormal proteins of a higher-order structure or proteins that are not normally modified accumulate in the ER lumen, and this can be observed as an expansion of the ER under TEM (Ishikawa et al., 2021). Since ER stress damage cells, cells are equipped with a system to avoid this, which is referred to as the unfolded protein response, wherein *CHOP* and *ATF6* expressions increase (Wu et al., 2007; Li et al., 2016). ER stress is observed in vEDS (Muller et al., 2012; Ishikawa et al., 2021). In this study, TEM showed no dilation of the ER in the normal control, but dilations of the ER were confirmed in the images of the disease control. Contrarily, in the present case, the expansion of the ER was not noticeable (Figure 4A). In real-time RT-PCR using mRNA extracted from the cultured fibroblasts, PCR was performed on four technical replicates of each mRNA sample, and the averages of the results were determined. *ATF6* and *CHOP* were highly expressed in the disease control when compared to the normal control; however, their expression levels in the present case were almost the same as those of the normal control (Figure 4B).

## Discussion and conclusion

According to the American College of Medical Genetics and Genomics (ACMG) guidelines (Richards et al., 2015), PM2 (not registered in the database) and PM4 (in-frame variant) corresponded to the patient’s variant. *In silico* analysis with multiple programs has been difficult to assess in in-frame variants. Since neither parent had symptoms of vEDS, this variant was presumably the *de novo* variant; however, the parent’s genes could not be confirmed. Therefore, the evaluation of the ACMG guidelines remains at “Variant of Unknown Significance (VUS).” However, the clinical findings were mild in the present case and were consistent with the symptoms caused by abnormalities in *COL3A1*, and a decreased collagen III was also confirmed *in vitro*. In the TEM analysis, morphological abnormalities of collagenous bundles were apparent when compared with those of the normal control. We attributed these findings to abnormalities in *COL3A1*, although VUS was assessed at the ACMG. Contrarily, this case interestingly showed no evidence of ER stress.

In vEDS cases with the commonly reported Gly mutations in the [Gly-X-Y] repeats or splice-site mutations, the aberrant peptide chains produced by the mutant alleles inhibit the formation of normal peptide triple helices. Practically, the expression level of collagen III is reduced to nearly 10% that of healthy people, which is known as a dominant negative effect (Mao and Bristow, 2001; Shimaoka et al., 2010; Malfait et al., 2017). In collagen fibers, the repeating sequence of [Gly-X-Y] is critically important for maintaining the triple helical structure; if even one of the three peptide chains has an abnormality in the repeating sequence, the triple helical structure cannot be maintained (Mao and Bristow, 2001). Adverse effects of pathogenic gene mutations do not manifest themselves only in protein synthesis processes. Moreover, aberrant peptide chains those fail to construct a triple helical structure due to protein folding in the ER accumulate in the ER (Malfait et al., 2017; Ishikawa et al., 2021), causing ER stress and consequent cell damage (Schroder and Kaufman, 2005).

Conversely, epidemiological studies have already shown that vEDS patients with nonsense mutation display a mild phenotype (Pepin et al., 2014). In nonsense mutations, the abnormal alleles stop producing the peptide chains, which is called haploinsufficiency. Therefore, the peptide chain produced is only from the normal allele, and the dominant negative effect is avoided. Theoretically, collagen III levels were reduced to 50% when compared to the normal control with less major detrimental effects. Furthermore, it is expected that the nonsense variant also has a reduced ER stress response because there is no accumulation of any aberrant peptide chains in the ER (Muller et al., 2012).

In patients with vEDS, although collagen III must be theoretically extremely low from birth, serious complications occur more frequently in adulthood and later, which are rarely seen during infancy (Malfait et al., 2017). Although the pathophysiology of vEDS is still not fully explained, it is considered that the various symptoms in vEDS are caused by a hybrid of two factors: the direct effect of the decrease in collagen III and damaged fibroblasts caused by ER stress (Ishikawa et al., 2021). However, hypothetically, if an in-frame mutation that does not affect the order of [Gly-X-Y] occurs, as in this case, the folding phenomenon in the ER would be mostly normal. Then, the triple helical structure can be maintained in the ER. The absence of an ER expansion might be an indication of avoidance of the dominant negative effects. On the other hand, the mutation in the present case involves the incorporation of two more [Gly-X-Y] residues into the helix. In the collagenous bundles, collagen III would be composed of peptide chains of different lengths (normal length and two [Gly-X-Y] residues as long length) in this case. Presumably, such collagenous bundles are unstable in the extracellular matrix and fragile outside the cell. In this case, the mildly reduced collagen expression, minor morphological abnormalities of the collagenous bundles, and near-normal ER stress support this hypothesis. The lack of ER stress would be associated with a mild phenotype. We have previously reported a case of a gene mutation that does not affect the [Gly-X-Y] repeats, which was a 9-bp deletion. That case was also confirmed to show a mild phenotype (Hayashi et al., 2020). Although there are a few reports of such gene mutations, which are in-frame duplications/deletions in multiples of nine, there is a possibility that such mild phenotypes may not cause serious symptoms. Moreover, patients with such cases may have not gone through genetic examinations at a medical institution due to overlooked vEDS. Thus, there may be undiagnosed patients.

Unfortunately, it is not easy to clearly determine whether the triple helical structures are composed of different lengths of peptide chains in combinations of wild-type and mutant alleles (not affecting the [Gly-X-Y] repetitions) in the extracellular matrix. Furthermore, there are many individual differences in ER stress responses due to the influence of epigenetics (Ramos-Lopez et al., 2018); thus, there is no conclusive evidence to conclude that just the absence of ER stress is associated with dominant negative avoidances. Therefore, this study has its limitations and cannot go beyond the hypothesis.

In conclusion, in the present study, our analyses has indicated that the identified mutations, which are in-frame duplications/deletions in multiples of nine, might underlie the pathogenesis observed in vEDS with a mild phenotype. Identifying the pathogenicity of this unique variant, which has morphological abnormalities in the collagenous bundles despite lower ER stress, is very important for elucidating the pathophysiology of vEDS in the future. The age at onset and severity of vEDS greatly vary regardless of the variant present among the patients, and it is important to investigate the role of ER stress in this clinical difference.

## Data Availability

The data sets for this article are not publicly available due to concerns regarding participant/patient anonymity. Requests o access the data sets should be directed to the corresponding author.
